# Synthesis of Non-Uniform Functionalized Amphiphilic Block Copolymers and Giant Vesicles in the Presence of the Belousov–Zhabotinsky Reaction

**DOI:** 10.3390/biom9080352

**Published:** 2019-08-08

**Authors:** Isadora Berlanga

**Affiliations:** 1 Department of Earth and Planetary Sciences and Origins of Life Initiative, Harvard University, 100 Edwin H. Land Bvld., Cambridge, MA 02138, USA; isadora.berlanga@ing.uchile.cl; Tel.: +56-2297-84795; 2 Department of Chemical Engineering, Biotechnology and Materials. FCFM, Universidad de Chile, Beauchef 851, Santiago 8370456, Chile

**Keywords:** giant vesicles, morphology control, amphiphilic block copolymers, Belousov–Zhabotinsky (BZ) reaction, synthetic design

## Abstract

Giant vesicles with several-micrometer diameters were prepared by the self-assembly of an amphiphilic block copolymer in the presence of the Belousov–Zhabotinsky (BZ) reaction. The vesicle is composed of a non-uniform triblock copolymer synthesized by multi-step reactions in the presence of air at room temperature. The triblock copolymer contains poly(glycerol monomethacrylate) (PGMA) as the hydrophilic block copolymerized with tris(2,2′-bipyridyl)ruthenium(II) (Ru(bpy)_3_), which catalyzes the BZ reaction, and 2-hydroxypropyl methacrylate (HPMA) as the hydrophobic block. In this new approach, the radicals generated in the BZ reaction can activate a reversible addition-fragmentation chain transfer (RAFT) polymerization to self-assemble the polymer into vesicles with diameters of approximately 3 µm. X-ray photoelectron spectroscopy (XPS) measurements demonstrated that the PGMA-*b*-Ru(bpy)_3_-*b*-PHPMA triblock copolymer is brominated and increases the osmotic pressure inside the vesicle, leading to micrometer-sized features. The effect of solvent on the morphological transitions are also discussed briefly. This BZ strategy, offers a new perspective to prepare giant vesicles as a platform for promising applications in the areas of microencapsulation and catalyst support, due to their significant sizes and large microcavities.

## 1. Introduction

Polymeric giant vesicles are micrometer-sized supramolecular structures consisting of a closed bilayer structure formed by self-assembling amphiphiles [1]. In comparison with those of phospholipids, the physical and chemical properties of polymer-based artificial vesicles can be optimized by introducing selected amphiphilic blocks and/or the variations in the molecular weight [2,3]. The chemical versatility of block copolymers allows for tuning of the membrane properties and surface functionalities of the vesicles [4,5]. In recent years, giant vesicles have received significant attention in a number of applications, such as microcapsules in drug and gene delivery systems [6,7], microreactors [8], and mimics for biological membranes [9,10,11,12]. For the successful implementation of giant vesicles into many of these applications, it is important to fabricate size-controlled vesicles loaded with certain desired functionalities. However, commonly used fabrication techniques typically produce polydisperse assemblies with broad size distributions, because vesicle formation depends significantly on non-equilibrium aspects of the assembly process [1]. Indeed, it is a challenging task to prepare uniform-sized vesicles with the desired diameter.

Recently, polymerization-induced self-assembly (PISA) via reversible addition-fragmentation chain transfer (RAFT) has become a powerful tool for the rational, efficient design and self-assembly of a wide range of block copolymers using a one-pot synthesis [13,14,15]. The resulting polymers can produce nanometer-sized morphologies at high concentrations (10–50% *w*/*w*) [16]. Their chemical composition, molecular weight and block length ratios can be manipulated directly by the reaction conditions to control the size and resulting morphology of the vesicles [17,18]. In general, the nature of morphologies in PISA reaction is affected by the solvent and the copolymer concentrations [19,20,21]. Depending of these factors, the available nanoscale morphologies include micelles, spheres, worms and vesicles [17]. In particular, stimulus-responsive block copolymers can be designed to undergo a morphological transition when exposed to an external stimulus such as light [22,23,24,25,26], temperature [15] or pH [27,28]. More recently, these reactions have been extended to produce micrometer-sized polymer vesicles using light by a photopolymerization-induced self-assembly (photo-PISA) [29,30,31]. 

Because of their micrometer size, the giant vesicles obtained by PISA are interesting in the area of biomimetics [32]. E. Yoshida has synthesized block copolymer giant vesicles employing the photoirradiated, nitroxide-mediated polymerization (photo-NMP) technique to initiate a PISA process [29,33,34,35]. The vesicles comprised an amphiphilic poly (methacrylic acid)-block-poly(methyl methacrylate-random-methacrylic acid) random block copolymer, PMAA-*b*-P(MMA-*r*-MAA), had some similarities with biomembranes, especially with regards to the bud separation [33] and fusion [29,35] that was caused by the appropriate external perturbations.

To further develop biomimetic membranes based on amphiphilic block copolymers, it is important to include fundamental functions and mechanisms in the artificial cell within the polymer vesicle. These include oscillatory, dynamic behaviors that occur in the compartmentalized system. The most widely known and studied chemical oscillating reaction is the Belousov–Zhabotinsky (BZ) reaction [36,37], which consists of the oxidation of an organic substrate (such as malonic or citric acid) by an oxidizing agent (bromate ion) in the presence of a strong acid and metal catalyst. Yoshida’s group has developed a class of biomimetic materials, called self-oscillating polymers, that can exhibit spontaneous changes with temporal or spatial periodicity in response to oscillatory chemical changes [38,39,40]. Typically, in this type of system, the linked polymer network participates in the BZ reaction through the metal catalyst, Ru(bpy)_3_, that is covalently bonded to the polymer chain. In the presence of the BZ reactants, the synthetic polymer undergoes spontaneous swelling/deswelling oscillations, producing the formation/fragmentation of giant vesicles [39,41,42]. However, the addition of an extra source of energy, such as ultra violet (UV) light [41] or high temperature, is required to initiate the polymerization, and the results indicate that the BZ reaction provides limited control over the vesicle size distribution. 

Recently, an alternative methodology has been developed to form giant vesicles by coupling PISA and oscillation chemistry at room temperature [43,44]. The radicals generated in the BZ reaction initiate the polymerization of amphiphiles, that leads to self-assembly and formation of nanoscale micelles which eventually transform into micrometer polymer vesicles. The resulting giant vesicles entrap the BZ reaction and autonomously makes them bleb and divide into small vesicles. These studies provide the possibility of using PISA to produce giant vesicles from amphiphilic block copolymers for the construction of artificial cells. 

In this study, we report on the morphological changes and diameter size distributions of polymer vesicles in the presence of the BZ reaction. With this purpose in mind, we present a new “multi-pot” method for preparing vesicles based on amphiphilic block copolymers via RAFT polymerization and in the presence of the BZ reaction. In this approach, a PISA process is not implemented, but giant vesicles are obtained at room temperature in the presence of oxygen. Moreover, it is conducted in the absence of stirring and external catalysts or initiators, which accommodates polymerization under biologically relevant conditions. To prepare the amphiphilic triblock copolymer, the ruthenium catalyst Ru(bpy)_3_ was first copolymerized with a hydrophilic block of poly(glycerol monomethacrylate) (PGMA). Then, the hydrophobic part, 2-hydroxypropyl methacrylate (HPMA), was attached and polymerized to the main chain, PGMA-*b*-Ru(bpy)_3_, in the presence of the BZ reaction. In the literature, there are many examples of PGMA-PHPMA diblock copolymers synthesized by PISA in aqueous media [27,45]. This approach provides access to nano-objects with different morphologies, such as spheres, worms and vesicles [17,46]. The HPMA monomer has the unusual property of being water-miscible (13% *w*/*w* at 25 °C) [47], but its polymer, PHPMA, is insoluble in water, which is an essential criterion for PISA. We use the radicals and ions generated in the BZ reaction to induce the RAFT polymerization of the hydrophobic part of the copolymer, which eventually self-assembles into giant vesicles. 

The effects of the solvent on the size distribution of the vesicles and their morphology were studied by using cryogenic scanning electron microscopy (Cryo-SEM) as well as field emission scanning electron microscopy (FE-SEM). To the best of our knowledge, this is (a) the first amphiphilic triblock copolymer synthesized using the radicals produced by the BZ reaction and is the first example of (b) self-assembly into giant vesicles at room temperature, in the presence of oxygen and in absence of stirring.

## 2. Materials and Methods 

### 2.1. Materials 

All chemicals were purchased at the highest available purity. Diethyl ether, anhydrous lithium bromide, *N*,*N*-dimethylformamide and HPMA (as a mixture of isomers) were purchased from Alfa Aesar (Ward Hill, MA, USA). Methanol, 4-cyano-4-(phenylcarbonothioylthio)pentanoic acid (CADB), sodium bromate, malonic acid, 2,2′-azobis(2-methylpropionitrile) (AIBN), ammonium hexafluorophosphate, dimethyl sulfoxide-*d6*, hexamethyldisilazane, ethanol and cis-Dichlorobis(2,2′-bipyridine)ruthenium(II) were purchased from Sigma-Aldrich (Haverhill, MA, USA). Ruthenium (4-vinyl-4′-methyl-2,2′-bipyridine)bis(2,2′-bipyridine)bis(hexafluorophosphate)) (Ru(bpy)_3_ monomer) was synthesized according to the previously reported method [48]. Nitric acid (6N) was purchased from BDH. 4-methyl-4′-vinyl-2,2′-bipyridine was purchased from Ark Pharm Inc. (Arlington Heights, IL, USA). Deuterium oxide was purchased from Cambridge Isotope Laboratories (Andover, MA USA). Glycerol monomethacrylate (GMA) was purchased from Polysciences Inc (Warrington, PA, USA). Aluminum oxide (basic, Brockmann I) for chromatography, 50–200 µm, 60 Å, was purchased from Acros Organics (West Chester, PA, USA). All products were used as received, except for GMA, which contains isomeric impurities and was purified by passing through an aluminum oxide column, as well as AIBN, which was purified by recrystallization in methanol. 

### 2.2. Synthesis and Characterization of Poly(Glycerol Monomethacrylate) 

The CTA (chain transfer agent) was CADB (58 mg, 0.21 mmol), the monomer, GMA (2 g, 12.48 mmol; target degree of polymerization (DP) = 60), and the initiator, AIBN (6.8 mg of 0.0416 mmol), were placed in a Schlenk flask and the atmosphere was replaced with nitrogen (N_2_). Then, 16 mL of anhydrous MeOH was added through the septum. The solution was stirred until all the CTA had dissolved. The Schlenk flask was placed in an ice bath and purged under N_2_ for 40 min at 0 °C. The RAFT polymerization was carried out at 70 °C for 18 h. The polymerization was quenched at 84% conversion by exposure to air, followed by cooling the reaction vessel in an ice bath. The crude PGMA homopolymer was purified three times by reprecipitation using methanol as a good solvent and diethyl ether as a poor solvent and dried 1 day under vacuum at room temperature. A pale pink solid was obtained. Yield: 1.1478 g (57.4%). Proton nuclear magnetic resonance (^1^H-NMR) studies indicated a mean degree of polymerization of 56 via end-group analysis (the integrated aromatic RAFT end-group signals at 7.1−7.4 ppm were compared to those assigned to the methylene protons at 1.8 ppm). DMF gel permeation chromatography (GPC) indicated a M_n_ of 14,500 g mol^−1^ and an M_w_/M_n_ of 1.4.

### 2.3. Synthesis and Characterization of the Poly(Glycerol Monomethacrylate)-Poly(Rris(2,2′-Bipyridyl)Ruthenium(II)) Diblock Copolymer

PGMA macro-CTA (109.66 mg, 0.0111 mmol), Ru(bpy)_3_ monomer (50 mg, 0.0555 mmol; target DP = 5) and AIBN (1 mL, 0.0022 mmol) were dissolved in methanol and placed in a Schlenk flask, and the atmosphere was replaced with nitrogen (N_2_). Then, 11 mL of anhydrous methanol was added through the septum. The solution was stirred until all of the CTA and Ru(bpy)_3_ had dissolved. The Schlenk flask was placed in an ice bath and purged under N_2_ for 40 min at 0 °C. The RAFT polymerization was conducted at 70 °C for 48 h to ensure complete monomer conversion. The polymerization was then quenched by exposure to air, cooled in an ice bath and dried overnight under vacuum at room temperature. A red solid was obtained. The structure of the polymer was characterized by ^1^H-NMR spectroscopy after it was dialyzed with water for 3 days and dried in a rotary Speed Vac (Thermo Fisher Scientific, Cambridge, MA, USA) evaporator at 60 °C. DMF GPC indicated a M_n_ of 19,300 g mol^−1^ and an M_w_/M_n_ of 1.2.

### 2.4. Preparation of the Calibration Curve to Determine the Amount of Poly(Tris(2,2′-Bipyridyl)Ruthenium(II)) Attached to the Polymer

The calibration curve was independently made from aqueous solutions of Ru(bpy)_3_PF_6_ with known concentrations. The absorbance spectra of the aqueous solutions of Ru(bpy)_3_PF_6_ were measured using UV-Vis spectroscopy. 

### 2.5. Synthesis and Characterization of the Non-Uniform Poly(Glycerol Monomethacrylate)-Poly(Tris(2,2′-Bipyridyl)Ruthenium(II))–Poly(2-Hydroxypropyl Methacrylate) Triblock Copolymer 

PGMA-*b*-Ru(bpy)_3_ (10 mg, 0.5% *w*/*w*) was dissolved in 1043.6 µL of distilled water and subjected to the BZ reaction ([MA] = 62.4 mM, [NaBrO_3_] = 84 mM, [HNO_3_] = 300 mM) at room temperature for 1 h. Then, 600 mg of HPMA (2-hydroxypropyl methacrylate) (30% *w*/*w*, target DP = 4160) was added to the solution for 23 h. Finally, the product was rotary evaporated at 60 °C for 90 min, purified three times by reprecipitation using MeOH as a good solvent and diethyl ether as a poor solvent and dried overnight under vacuum at room temperature. A yellow solid was obtained. The structure of the polymer was characterized by ^1^H-NMR spectroscopy. DMF GPC indicated a M_n_ of 49,700 g mol^−1^ and an M_w_/M_n_ of 4.3. The composition ratio of the triblock copolymer was determined to be PGMA/(Ru(bpy)_3_)/PHPMA = 56:1:274 by ^1^H-NMR, UV−vis and GPC measurements.

We prepared a control experiment (without the BZ reaction) according to the following procedure: 10 mg of PGMA-*b*-Ru(bpy)_3_ (0.5% *w*/*w*) was dissolved in 1043.6 µL of distilled water and was left at room temperature for 1 h. Then, 600 mg of HPMA (30% *w*/*w*) was added to the solution for 23 h. The products were evaporated at 60 °C for 90 min and dried overnight under vacuum at room temperature. Yield: 12.5 mg. The morphology of the product was characterized by SEM. 

### 2.6. X-ray Photoelectron Spectroscopy Sample Preparation

To analyze the samples of the NaBrO_3_ starting material and the PGMA-*b*-Ru(bpy)_3_-*b*-PHPMA triblock copolymer, 2 mg of a solid was placed on a conductive doubled-sided adhesive carbon tape to form a very thin film on an aluminum support. Then, the sample was introduced into an ultrahigh-vacuum chamber.

### 2.7. Sample Preparation for Field Emission Scanning Electron Microscopy and Focused Ion Beam

A total of 2 mg of the PGMA-*b*-Ru(bpy)_3_-*b*-PHPMA triblock copolymer was dissolved in 100 µL of methanol. Then, we dropped 60 µL of the PGMA-*b*-Ru(bpy)_3_-*b*-PHPMA solution on a micro glass coverslip (Ted Pella Inc., Redding, CA, USA), waited 7 min and washed it three times with hexamethyldisilazane to dry the sample. Finally, the micro glass was attached to the conductive double-sided adhesive carbon tape and sputter-coated with a thin layer of platinum and palladium before imaging. We also prepared control samples according to the following procedure. We prepared the samples for solvent effect studies as follows: 2 mg of PGMA-*b*-Ru(bpy)_3_-*b*-PHPMA was dissolved in water/methanol mixtures consisting of 0, 70, or 100% methanol. Then, we dropped 60 µL of a PGMA-*b*-Ru(bpy)_3_-*b*-PHPMA solution onto a micro glass coverslip, waited 7 min and washed it three times with hexamethyldisilazane to dry the sample. Finally, the micro glass was attached to the conductive double-sided adhesive carbon tape and sputter-coated with a thin layer of platinum and palladium before imaging.

### 2.8. Sample Preparation for Cryogenic Scanning Electron Microscopy 

A total of 2.5 mg of PGMA-*b*-Ru(bpy)_3_-*b*-PHPMA was dissolved in a water/methanol mixture (1/4, *v/v*). After that, we dropped 2 µL of a PGMA-*b*-Ru(bpy)_3_-*b*-PHPMA solution onto a copper grid. The sample was then frozen by liquid nitrogen and characterized by cryo-SEM run at an accelerating voltage of 2 kV.

### 2.9. Polymer Characterization 

^1^H-NMR spectroscopy. All NMR spectra were recorded using a 500 MHz Varian Unity/Inova500 spectrometer (Laukien-Purcell Instrumentation Center, Cambridge, MA, USA)

The optical transmittance of PGMA-*b*-Ru(bpy)_3_ diblock copolymer the solution was measured by using a UV-Vis spectrophotometer (Unico S2100 UV-Vis spectrophotometer, Cambridge, MA, USA). 

The redox oscillations of the reaction solution were measured by a combination redox electrode (MI-800-410, Microelectrodes, INC., Bedford, NH, USA) connected to a benchtop meter (Sper scientific 860031 benchtop pH/mV meter, Scottsdale, AZ, USA). This benchtop meter was also connected to a computer for data collection and storage. 

Gel permeation chromatography (GPC). The molecular weights and polydispersities were determined by a DMF GPC setup operating at 50 °C. The system used one PL1113-6300 ResiPore 7.5 × 300 mm column with HPLC-grade DMF (stabilized with 0.05 M LiBr) at a flow rate of 1.0 mL/min. Calibration was conducted using a series of eight nearly monodisperse poly(methyl methacrylate) standards (M_n_ = 960–1568000 g mol^−1^). Chromatographs were analyzed using Agilent GPC/SEC software.

X-ray photoelectron spectroscopy (XPS). Spectra were obtained using a Thermo Scientific K-Alpha XPS photoelectron spectrometer with monochromatic Al K-α X-ray radiation (1.49 kV at a base pressure of ~10^−9^ Torr). The binding energies were corrected by referencing the C(1s) binding energy to 284.5 eV.

Field emission scanning electron microscopy measurements were performed using a Zeiss Supra 55VP scanning electron microscope with an accelerating voltage of 3 kV using an SE detector. Chemical characterization was conducted by energy dispersive X-ray spectroscopy (EDXS). Elemental composition was determined using electron beam accelerating voltages of 10 kV. 

Cryogenic-SEM and focused ion beam (FIB) images of vesicles were acquired using a Zeiss NVision (Center for Nanoscale Systems Harvard University, Cambridge, MA, USA) 40 field emission scanning electron microscope at acceleration voltages of 3 kV. 

## 3. Results

**Scheme 1:**
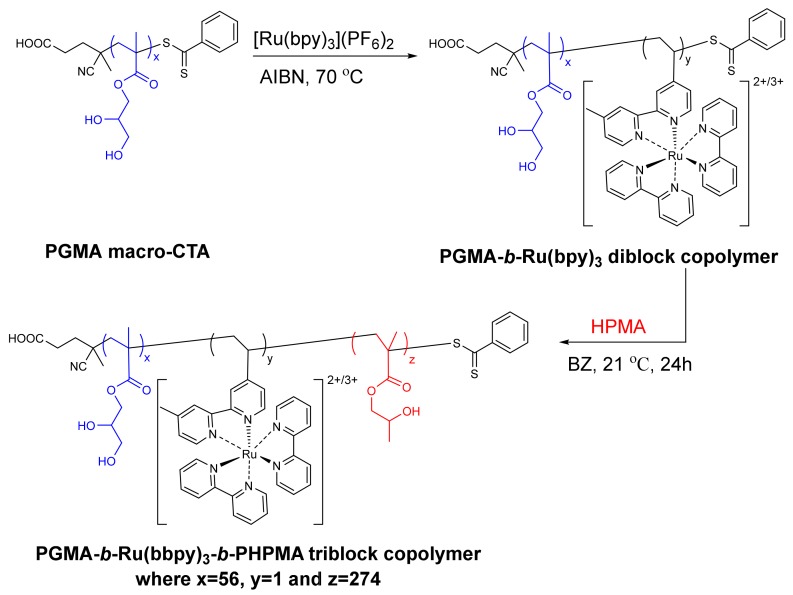
Schematic illustration of the synthesis of poly(glycerol monomethacrylate)-poly(tris(2,2′-bipyridyl)ruthenium(II)) –poly(2-hydroxypropyl methacrylate) (PGMA-*b*-Ru(bpy)_3_-*b*-PHPMA) triblock copolymer vesicles.

The procedure consists of three separate steps (Scheme 1). The first step involves the synthesis of the hydrophilic part, PGMA macro-CTA, followed by a second step where it is polymerized with the Ru catalyst to yield a PGMA-*b*-Ru(bpy)_3_ diblock copolymer. Both reactions were prepared by a thermally initiated RAFT polymerization. Finally, the Ru(bpy)_3_-functionalized diblock copolymer was polymerized with the hydrophobic block, consisting of HPMA, using the radicals generated by the host BZ reaction. During the BZ redox oscillations of the transition-metal catalyst, enough malonyl (${\mathrm{MA}}^{\cdot }$) and bromine dioxide (${\mathrm{BrO}}_{2}^{\cdot }$) radicals were generated to polymerize the third block of the triblock copolymer. We observed and studied the oscillating redox potential.

In more detail, in the first step we prepared the stabilizer block, a PGMA macro-CTA homopolymer, using AIBN as an initiator and CADB as a RAFT chain transfer agent in methanol at 70 °C to generate a hydrophilic, narrowly dispersed PGMA macro-CTA block (M_w_/M_n_ = 1.4) (see Supplementary Figures S1 and S2). A small molecular weight shoulder was observed, that could be attributed to some degree of termination by combination, which is the dominant mechanism for acrylic polymers [49]. It has been previously reported that the molecular weight shoulder frequently occurs in RAFT polymerizations at high monomer conversions due to bimolecular termination reactions of the growing polymer chains [50]. This general phenomenon is observed when using the RAFT method, due to the presence of several radicals in the system during the polymerization. ^1^H-NMR analysis indicated a mean degree of polymerization of 56 for this PGMA macro-CTA.

This PGMA macro-CTA homopolymer was then used for the RAFT polymerization of Ru(bpy)_3_ to obtain the PGMA-*b*-Ru(bpy)_3_ diblock copolymer. ^1^H-NMR spectroscopy confirmed the presence of Ru(bpy)_3_ complexes attached to the polymer by the proton signals assigned to bipyridine rings between 7 and 9 ppm (see Supplementary Figure S3). To estimate the ratio of Ru(bpy)_3_ in the polymer chain quantitatively, we measured the absorbance of the PGMA-*b*-Ru(bpy)_3_ solution at 457 nm by UV-vis spectrophotometry and the ratio was calculated by means of a calibrated curve (see Supplementary Figure S4). The conjugation ratio of Ru(bpy)_3_ to PGMA macro-CTA was calculated to be 1 mol%. Empirically, we find that this 1 mol% of Ru(bpy)_3_ is sufficient to produce giant vesicles in the presence of the BZ reaction. Furthermore, GPC analysis in DMF indicated a shift of the molecular weight distribution to higher molecular weight (Mn = 19300 g mol^−1^) (see Supplementary Figure S2). The presence of a weak high molecular weight shoulder was also formed by the effect of termination by combination reaction, which was not completely avoidable by the chosen reaction conditions. However, this shoulder does not substantially broaden the molecular weight distribution, as well as the low polydispersity (M_w_/M_n_ = 1.2), suggesting that RAFT polymerization was conducted in a well-controlled way with a high chain transfer efficiency for PGMA-*b*-Ru(bpy)_3_ diblock copolymer. Thus, it does not show effects in the subsequent synthesis steps. 

To further characterize this Ru-containing polymer, we studied the oscillation profile of the redox potential during the BZ reaction for the PGMA-*b*-Ru(bpy)_3_ diblock copolymer solution at 20 ± 1 °C. As the polymer was dissolved in the solution containing the BZ substrates, the ruthenium catalyst changed its redox state. In particular, the transition-metal complex Ru(bpy)_3_ displays a significantly different solubility in water between its oxidized and reduced states, which leads to the aggregation/disaggregation of the polymer chains. This generates periodic episodes of solubility/insolubility in the polymer that further influence the profile of the redox potential over time of the BZ solution (Supplementary Figure S5). At the beginning of the oscillations, the average of the BZ redox potential profile in the presence of the diblock copolymer gradually decreases because the size of the polymer aggregate in the reduced state increases with time. After this reduction, the average redox potential increased again due to the dissociation of the polymer aggregates produced by the repulsive force between the positively charged ruthenium catalysts attached to the polymer. This phenomenon has also been observed in polymers with positive charges such as methacrylamidopropyltrimethylammonium chloride (MAPTAC) during the BZ reaction [51]. Note also that the cationic moiety of MAPTAC-containing polymer chains generates the autonomous dissociation of the large polymer aggregates as a result of the electrostatic repulsive force between these MAPTAC components. It is serendipitous that our polymer, PGMA-*b*-Ru(bpy)_3_, has a slower oscillation compared to other polymers (approximately 11 h) [52]. These longer oscillations can be attributed to the presence of the positive charges, which prevent aggregation while in the reduced state. 

**Figure 1:**
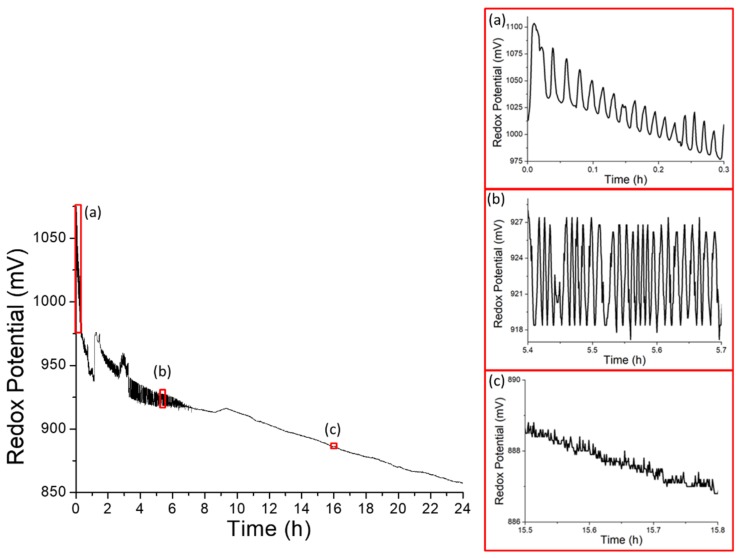
Oscillatory profile of the Belousov–Zhabotinsky (BZ) redox potential at 20 ± 1 °C for the synthesis of PGMA-*b*-Ru(bpy)_3_-*b*-PHPMA triblock copolymer vesicles. Zoomed areas shows a detail of the oscillation of redox potential at different times: (**a**) 0 h, (**b**) 5 h and (**c**) 15 h. The solution contains MA (62.4 mM), NaBrO_3_ (84 mM), and HNO_3_ (300 mM).

After the above synthesis of the PGMA-*b*-Ru(bpy)_3_ prepolymer, we proceeded to perform its block copolymerization with the hydrophobic block consisting of HPMA. The block copolymerization was performed at 20 ± 1 °C for 24 h in aqueous solution during the BZ reaction and in the dark, to avoid the contribution of $\mathrm{Ru}{\left(\mathrm{bpy}\right)}_{3}^{\cdot }$ radicals to polymerization. The reaction was investigated by redox potential measurements, in open air, that were recorded over time (Figure 1). Contrary to the control experiment (synthesis without the BZ components; see Supplementary Figure S6), as soon as the PGMA-*b*-Ru(bpy)_3_ diblock copolymer was added to the other components of the BZ reaction, redox oscillations began to occur and were recorded (see Figure 1a and, in the Supplementary Figure S7a). As the number of chemical oscillations increased, the amplitude of the redox potential of the polymer oscillation decreased. This is because, as we mentioned above, this BZ reaction takes more time to transform from the reduced state to the oxidized state than it does to go from the reduced to the oxidized state. This, together with the low solubility of the polymer in the reduced state, leads to polymer aggregation. As the aggregation/disaggregation of the polymer in the BZ reaction repeats, the size of the polymer aggregate in the reduced state increases, and the color remains red due to the hydrophobicity of the polymer, which tends to be in the reduced state. 

After 1 h, the oscillatory behavior stabilized, and we considered the number of malonyl radicals (${\mathrm{MA}}^{\cdot }$) and bromine dioxide radicals (${\mathrm{BrO}}_{2}^{\cdot }$) produced during the BZ reaction to be sufficient to start the polymerization of the hydrophobic block with 30% *w*/*w* of HPMA (see Supplementary Figure S7b). The addition of HPMA to the PGMA-*b*-Ru(bpy)_3_ diblock copolymer does not show any periodic oscillatory response, and the Ru(bpy)_3_ remains in the reduced state (red) during the course of the reaction, indicating a lower tendency towards oxidation in this medium. Observations show that after 4 h (see Figure 1b and Supplementary Figure S7c) the HPMA affects the system in two ways: (1) it creates an induction period for the start of the cycling of the redox potential, and (2) it produces non-periodic fluctuations accompanied by polymer phase separation. These two effects are most likely caused by the interaction between HPMA and the BZ intermediates, e.g., bromination of the HPMA double bond by a HOBr or a bromine (Br_2_) addition reaction. Pojman et al. reported that acrylonitrile polymerized periodically under the effects of the BZ reaction [53]. The resultant polyacrylonitrile was partially brominated via reactions with HOBr and Br_2_ [54]. In comparison with this previous result, it is reasonable to assume that bromine dioxide radicals (${\mathrm{BrO}}_{2}^{\cdot }$) are able to react with the polymer (P) to produce the radical species $P^{\cdot }$ during the BZ reaction:

(1) $$P+{\mathrm{BrO}}_{2}^{\cdot }\to P^{\cdot }+{\mathrm{HBrO}}_{2}.$$

The combination of ${\mathrm{BrO}}_{2}^{\cdot }$ with $P^{\cdot }$ can form P-BrO_2_ species at the end of the polymer chain, according to the reaction:

(2) $$P^{.}+{\mathrm{BrO}}_{2}^{\cdot }\to {\mathrm{PBrO}}_{2}.$$

The bromous ester end group hydrolyzes in the acidic medium of the BZ reaction to leave an alcohol group on the end of the polymer, analogous to the reaction between ${\mathrm{BrO}}_{2}^{\cdot }$ and malonyl radical (${\mathrm{MA}}^{\cdot }$) to produce tartaric acid (MA-OH) [54]

(3) $$\mathrm{POBrO}+H^{+}\to \mathrm{POH}+{\mathrm{HBrO}}_{2}.$$

We suggest that the ${\mathrm{BrO}}_{2}^{\cdot }$ can also abstract a hydrogen from the terminal $P^{\cdot }$, thus leaving a vinyl group as an end group. The vinyl group could then be brominated by either HOBr or Br_2_:
(4)
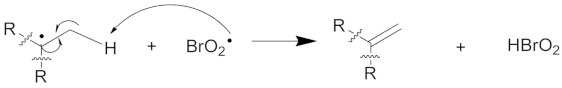

(5)
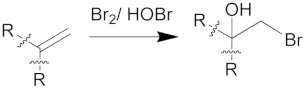


**Figure 2:**
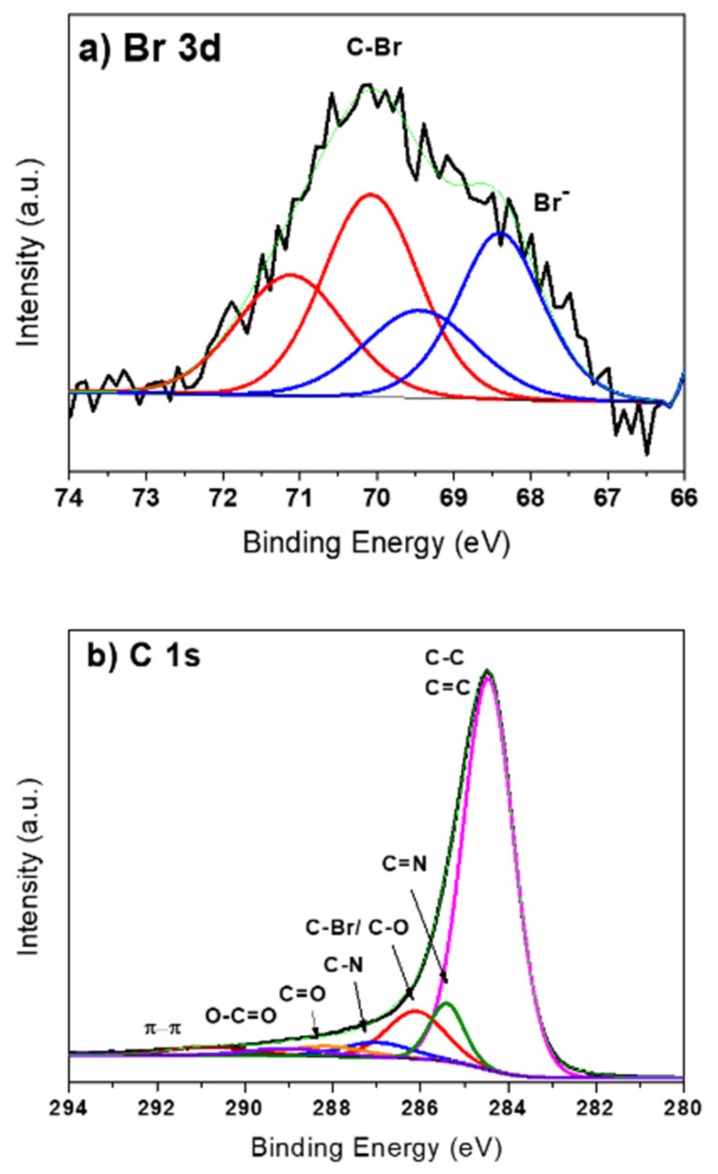
High-resolution X-ray photoelectron spectroscopy (XPS) spectra of Br 3d (**a**) and C 1s (**b**).

Consequentially, after 9 h (see Figure 1c and Supplementary Figure S7d–f) a decrease in the oscillation periods was observed, which could be due to the consumption of the HOBr and Br_2_ species through reactions with PHPMA. To confirm this mechanism, the resulting yellow, milky precipitate was purified and characterized by X-ray photoelectron spectroscopy (XPS), ^1^H-NMR and GPC. XPS was used to confirm the bromination of PGMA-*b*-Ru(bpy)_3_-*b*-PHPMA. There are two ways of determining the bromine composition by XPS: from resolution of the bromine peaks or of the carbon peaks (Figure 2). The high-resolution XPS spectrum of the doublets with the larger Br 3d 5/2 peaks located at 70.1, and 68.4 eV due to C-Br, and Br‾, respectively, with a constant fwhm of 1.5 eV (Figure 2a) [55,56]. According to the control experiment, the peak at 68.4 eV is associated with NaBrO_3_ impurities (see Supplementary Figure S8). The relative fractions of C-Br and Br‾ are 57 and 43%, respectively. Additionally, the C-Br bond signal can also be due to bromomalonic acid (BrMA), which is generated by the reaction of HOBr and Br_2_ with MA and enol groups, respectively. However, the Br 3d peak is not sufficient to distinguish between the HOBr, Br_2_ and BrMA species.

Additionally, as shown in Figure 2b, the high-resolution C 1s spectra were resolved into 6 peaks. C–C (284.5 eV), C=N (285.4 eV), C–Br (286.1 eV), C=O (287 eV), C–N (288.2 eV), O=C–O (289 eV), and a π–π* shake-up satellite peak (290.8 eV) were observed, and the signal of C–Br overlapped with of C–O. The small peak at 289 eV can be attributed to the presence of COOH edge groups or carboxylic ester groups, which indicates that the amount of BrMA in the polymer is insignificant (less than 3%). These results confirm that PGMA-*b*-Ru(bpy)_3_-*b*-PHPMA triblock copolymer was indeed brominated by HOBr and Br_2_. Furthermore, the O 1s high-resolution spectra corroborates the presence of C=O (531.3 eV), C–O (532.5 eV), –OH (533.1 eV), H_2_O (534.6 eV) and Na Auger KLL (536.8 eV) (see Supplementary Figure S9b). However, the peaks from ruthenium could not be detected (see Supplementary Figure S9c). This has also been noticed by other researchers and attributed to the lower loading of the complex, or to it overlapping with the C 1s peak [57]. The N 1s XPS spectrum displayed two peak components at 399.9 and 398.5 eV, due to C=N and C–N of bipyridine, which confirmed the presence of the ruthenium complex in the synthesized PGMA-*b*-Ru(bpy)_3_-*b*-PHPMA triblock copolymer (see Supplementary Figure S9d). 

^1^H-NMR spectra recorded for PGMA-*b*-Ru(bpy)_3_-*b*-PHPMA triblock copolymer did not include any prepolymer due to the purification by precipitation in methanol (see Supplementary Figure S10). Although all the signals associated with PGMA macro-CTA and PHPMA are clearly observed in the spectra, the Ru(bpy)_3_ signals are difficult to see due to the small amounts attached to the triblock copolymer. However, its presence in the polymer was corroborated by UV-vis spectroscopy: The shoulder near 461 nm can be attributed to the metal-to-ligand charge transfer state (d– π*) (see Supplementary Figure S11). Moreover, ^1^H-NMR analysis was somewhat complicated by overlapping peak integrals. The HPMA conversion was estimated to be ∼20% based on the signal intensity of the methyl protons at 1.1 ppm for the resulting copolymer and at 5.7–6.1 ppm for the remaining unreacted HPMA. As mentioned above, the resulted milky-yellow precipitate of the triblock copolymer confirms the high DP of PHPMA, 274, which became more difficult to study in water. This estimation of the conversion was consistent with DMF GPC analysis of obtained for the PGMA-*b*-Ru(bpy)_3_-*b*-PHPMA (see Supplementary Figure S2). Compared to that of the PGMA macro-CTA, the GPC traces obtained for the triblock copolymer were shifted to higher and broader molecular weight distributions (M_n_ = 49700 g mol^−1^, M_w_/M_n_ = 4.3). Due to the high molecular weight and polydispersity, the terminology non-uniform for describing PGMA-*b*-Ru(bpy)_3_-*b*-PHPMA triblock copolymer should be considered [58]. The low conversion and high polydispersity of the triblock copolymer can be attributed to the BZ reaction conditions (i.e., the absence of an initiator in the reaction). In comparison to the thermally initiated PGMA-PHPMA aqueous dispersion polymerizations reported in the literature [27,45], these results confirms that the rate of radicals generated by the aqueous BZ reaction is lower. However, the rate is high enough to synthesize giant vesicles based on the amphiphilic triblock copolymers. It should be note, that to produce giant vesicles of amphiphilic block copolymers based on PHPMA, high molecular weight distributions and polydispersities are required [59]. This suggests some loss of RAFT control under these conditions. In principle, the relatively high viscosity of the reaction mixture combined with no stirring conditions, may retard diffusion of the macro-CTA, but further studies are clearly warranted. Additionally, the HPMA monomer is known to undergo slow transesterification during storage at ambient temperatures, generating small impurities of a dimethacrylate species that can lead to greater levels of cross-linking during the RAFT polymerization of HPMA [17,60]. Thus, the homopolymerization of HPMA by the BZ reaction can also induce high polydispersities. Moreover, a high molecular weight shoulder becomes more prominent when the HPMA block is added. This result is quite similar to those reported for branched PHPMA syntheses involving the statistical copolymerization of HPMA with a divinyl comonomer using aqueous dispersion polymerizations [61]. 

**Figure 3:**
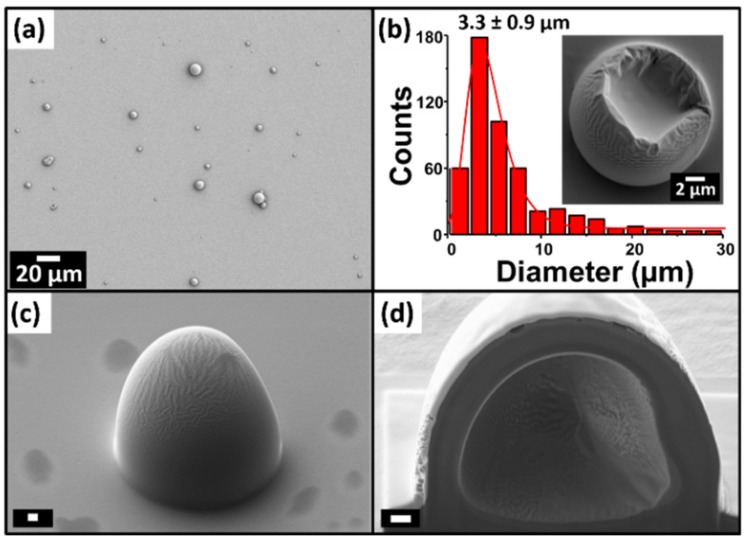
Field emission scanning electron microscopy (FE-SEM) images of PGMA-*b*-Ru(bpy)_3_-*b*-PHPMA triblock copolymer vesicles (**a**). Histogram of the vesicle diameters based on the FE-SEM measurements (inset: magnified SEM image of a single vesicle) (**b**). FE-SEM images before (**c**) and after (**d**) milling (scale bar = 1 µm).

FE-SEM of the PGMA-*b*-Ru(bpy)_3_-*b*-PHPMA triblock copolymer showed the presence of vesicles at 0.5% *w*/*w* in methanol (Figure 3a and Supplementary Figure S12). While the control experiment (synthesis without the BZ components; Supplementary Figure S13) give clean surfaces with and small deposits of unorganized material, the adsorption of the PGMA-*b*-Ru(bpy)_3_-*b*-PHPMA triblock copolymer in methanol solution shows vesicles. Analysis of the vesicles showed diameter distributions of 3.3 ± 0.9 µm (based on statistics performed on 500 vesicles) (Figure 3b). The fit shown in Figure 3b obtains a higher *R*-squared value (0.97478) by using an Extreme distribution of Doane’s formula [62]. The distribution of the diameters of the vesicles was highly skewed towards higher diameters, with a very pronounced peak at 3.3 µm, indicating that a large proportion of vesicles need a minimum diameter to begin growing. We observed the coexistence of spherical vesicles with a few elliptical vesicles and some apparent fusions, suggesting that perhaps the formation of the membranes resulted from the fusion of vesicles (see Supplementary Figure S14). The number of particles deposited onto the glass surface after 7 min showed that the coverage was 12 ± 2 particles per 500 µm^2^.

As we mentioned above, according to the XPS results, the PGMA-*b*-Ru(bpy)_3_-*b*-PHPMA triblock copolymer is brominated with the bromine from the NaBrO_3_ salt. The bromine attached to the triblock copolymer generates a high concentration of Na^+^ ions inside the vesicles. Consequently, the concentration inside the vesicles is higher than that which is outside, and the osmotic pressure inside of the vesicles increases. The osmotically induced flow of solvent into the vesicles causes the volume of the vesicles to swell to several micrometers. We also observed that, in vesicles with larger diameters (up to 10 µm), the increase in the internal osmotic pressure sometimes induced the vesicle to explode, probably due to increased lateral tension in a region of the membrane containing some defects.

A SEM equipped with a focused ion beam (FIB) was used to study the finer details of the vesicles. The well-formed vesicles of PGMA-*b*-Ru(bpy)_3_-*b*-PHPMA were subjected to FIB milling using a gallium ion beam. The selected portion of the vesicles was sliced out and to reveal a hollow interior (Figure 3c,d, and Supplementary Figure S15). Considerable variation is observed in the wall thickness of the spherical vesicles, with diameters ranging from 100 (few) to 2 μm. These observations suggest that multi-layer vesicles can be generated. However, this methodology did not provide any insight into the laminar organization of the resulting spherical vesicles. As we mentioned above in the GPC analysis, the polydispersity of the PGMA-*b*-Ru(bpy)_3_-*b*-PHPMA triblock copolymer can be attributed to the presence of homopolymerization of the hydrophobic block consisting of HPMA. The wall thickness can also be related to the presence of a polymer network inside the membrane. The PHPMA produced by the cross-linking of the hydrophobic block inside the polymer membrane enhances the stability of the vesicle structure, resulting in the formation of large vesicles.

**Figure 4:**
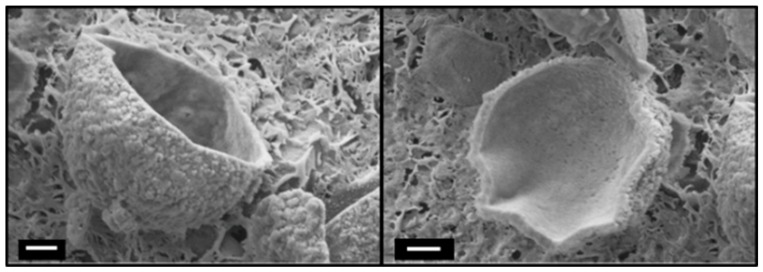
Cryo-SEM images of the PGMA-*b*-Ru(bpy)_3_-*b*-PHPMA triblock copolymer vesicles in a water/methanol solution (scale bar = 1 µm).

Cryo-SEM analysis of the 1.8% *w*/*w* PGMA-*b*-Ru(bpy)_3_-*b*-PHPMA triblock copolymer in a water/methanol solution (1/4, *v/v*) also reveals the presence of vesicles (Figure 4 and see Supplementary Figure S16). The mixture of solvents induces the formation of vesicles in the lower diameter range (less than 7 µm) with rough surfaces and roughly homogeneous dimensions. Analyzing the cross-section shows that this mixture of methanol and water produces thick walls in the range of 200–400 nm, with no observed multilayer structure. This difference in the thickness of the membrane can be attributed to the presence of water in the solution, which affects the final copolymer solubility, and thus, the resulting vesicle morphology. 

**Figure 5:**
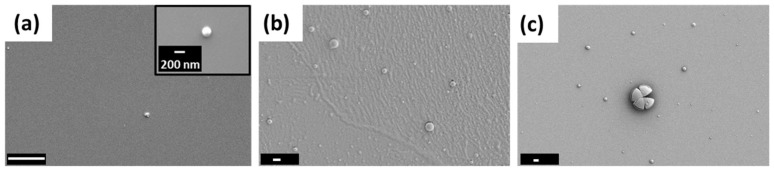
FE-SEM images of the PGMA-*b*-Ru(bpy)_3_-*b*-PHPMA triblock copolymer prepared in different water/methanol solutions: water (**a**), 70% methanol (**b**), and methanol (**c**). Scale bar = 2µm. The inset shows one micelle at higher magnification.

To test this hypothesis, we prepared a new set of experiments using different water/methanol mixtures. The obtained vesicle sizes are shown in Figure 5. FE-SEM images show that the type of solvent used has a strong effect on the size and morphology of the vesicles. We can clearly see that the size of triblock copolymer vesicles becomes larger with increasing methanol concentrations. Figure 5a shows that, in water, small micelles (100–200 nm in diameter) and low densities were observed. For 70% methanol, a mixed phase comprising micelles and vesicles is obtained (Figure 5b), while predominantly vesicles are observed in methanol. Increasing the methanol concentration further leads to an increase in the size and density of the vesicles. As shown in Figure 5c, vesicles prepared in methanol were larger and had higher densities than those prepared with 70% methanol. These results can be explained as follows: the hydrophilic block PGMA is more soluble in methanol than in water and, therefore, we propose that the PGMA will be better solvated with increasing amounts of methanol, which will cause a stretching of the chains, and thus the vesicle size will grow as the wall expands. However, more studies will be required in order to gain a better understanding of the morphological transition observed under these conditions.

## 4. Conclusions

In conclusion, we report here the first preparation of giant micron-size vesicles composed of a PGMA-*b*-Ru(bpy)_3_-*b*-PHPMA triblock copolymer by the BZ reaction. We followed and studied the oscillating profile of the reaction by redox potential measurements. We first analyzed the oscillating profile of the PGMA-*b*-Ru(bpy)_3_ diblock copolymer under the BZ reaction and then analyzed the addition of HPMA to the polymer. HPMA can play two roles in the system: bromination of the polymer and the generation of stable, large vesicles produced by the cross-linking of the PHPMA inside the membrane. We report a reproducible and controllable vesicle size between 0.8 and 30 μm with a modal population at 3.3 μm, which is well fitted by Extreme distribution. Moreover, we used the solvent composition to control the vesicle size and density. By preparing PGMA-*b*-Ru(bpy)_3_-*b*-PHPMA triblock copolymers in different ratios of water/methanol mixtures, we learned that smaller vesicles can be obtained by increasing the water content in the solvent mixture. The ability of the solvent composition to control the vesicle size and density is due to the hydrophilic block, PGMA, whose solubility in methanol resulted a stretching of the chains to form larger vesicles. Our next interest is to study the morphologies of amphiphilic triblock copolymers and their phase behavior in the presence of the BZ reaction, which will be presented in the future. This BZ-based strategy opens up a wide range of possibilities for the synthesis and design of new amphiphilic triblock copolymers that form giant vesicles with controlled sized distributions. The ability to reproducibly generate pure giant vesicles is expected to have important implications for the encapsulation of guest molecules, with potential applications in drug delivery and catalysis.

## Supplementary Materials

The following are available online at https://www.mdpi.com/2218-273X/9/8/352/s1, Figure S1: ^1^H-NMR spectra of the PGMA macro-CTA homopolymer in D_2_O. Figure S2: DMF GPC curves obtained for a PGMA macro-CTA (black) and the corresponding PGMA-*b*-Ru(bpy)_3_ diblock (red) and PGMA-*b*-Ru(bpy)_3_-*b*-PHPMA triblock copolymers (blue). Figure S3: ^1^H-NMR spectra of the PGMA-*b*-Ru(bpy)_3_ diblock copolymer in d6-DMSO. Figure S4: Calibration curve to determine the amount of Ru(bpy)_3_ conjugated to the polymer PGMA macro-CTA. Figure S5: Oscillation profiles of redox potential at 20 °C for 0.5% *w*/*w* PGMA-*b*-Ru(bpy)_3_ solutions. Figure S6: Oscillation profiles of redox potential for control experiment. Figure S7: Oscillation profiles of redox potential for the synthesis of PGMA-*b*-Ru(bpy)_3_-*b*-PHPMA under the BZ reaction at different times: (a) 0h, (b) 2h, (c) 5h, (d) 10h, (e) 17h, (f) 24h. Figure S8: XPS spectra of NaBrO_3_: a wide scan spectra (survey) (a) and Br 3d core level (b). Figure S9: XPS spectra of a wide scan spectra (survey) (a), O 1s(b), Ru 3p(c), and N 1s (d), core level spectra of PGMA-*b*-Ru(bpy)_3_-*b*-PHPMA triblock copolymer. Figure S10: ^1^H-NMR spectra of the PGMA-*b*-Ru(bpy)_3_-*b*-PHPMA triblock copolymer in DMSO-d6. Figure S11: UV-visible absorption spectra of PGMA-*b*-Ru(bpy)_3_-*b*-PHPMA triblock copolymer in a methanol solution. Figure S12: FE-SEM images of PGMA-*b*-Ru(bpy)_3_-*b*-PHPMA triblock copolymer vesicles. Figure S13: FE-SEM image of control experiment (a). A zoomed area shows a disorganized material (b). Figure S14: FE-SEM images shows examples of the morphology of fusing vesicles, Figure S15: FE-SEM images of PGMA-*b*-Ru(bpy)_3_-*b*-PHPMA triblock copolymer vesicles before (a and c) and after milling (b and d). Figure S16: Cryo-SEM images of PGMA-*b*-Ru(bpy)_3_-*b*-PHPMA triblock copolymer vesicles in a water/methanol mixture solution.
