# Supplementary material for: Synthesis of Non-Uniform Functionalized Amphiphilic Block Copolymers and Giant Vesicles in the Presence of the Belousov–Zhabotinsky Reaction
Source: Biomolecules. 2019 Aug 8;9(8):352. doi: 10.3390/biom9080352 (PMC6723531; doi:10.3390/biom9080352)
Supplement: Supplementary file 1 [file biomolecules-09-00352-s001.pdf]

SUPLEMENTARY INFORMATION

Synthesis of Non-Uniform Functionalized Amphiphilic Block Copolymers and Giant Vesicles in the Presence of the Belousov-Zhabotinsky Reaction

Isadora Berlanga [1,2]

[1] *Department of Earth & Planetary Sciences and Origins of Life Initiative, Harvard University, 100 Edwin H. Land Bvld Cambridge, MA 02138 (USA).*

[2] Department of Chemical Engineering, Biotechnology and Materials. FCFM, Universidad de Chile, Beauchef 851, Santiago, Chile.

E-mail: Isadora.berlanga@ing.uchile.cl

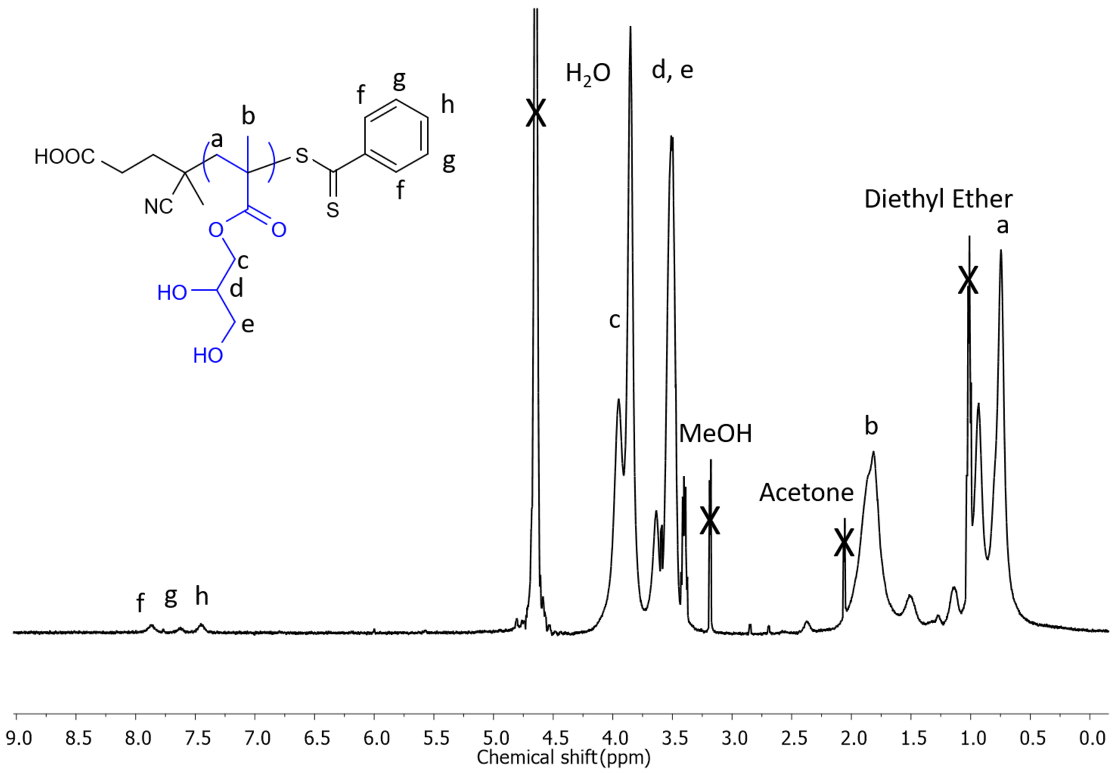

Figure 1. ^{1}H-NMR spectra of the PGMA macro-CTA homopolymer in D_2O.

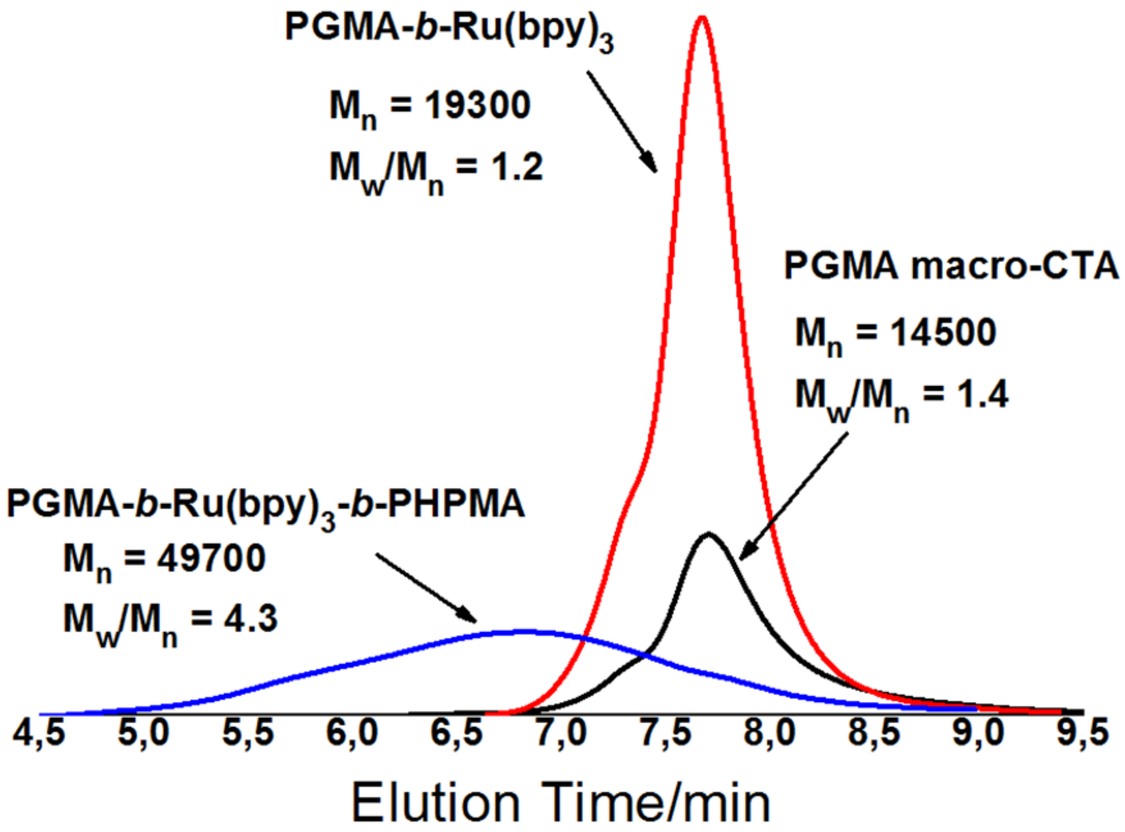

Figure S2. DMF GPC curves obtained for a PGMA macro-CTA (black) and the corresponding PGMA-*b*-Ru(bpy)$_3$ diblock (red) and PGMA-*b*-Ru(bpy)$_3$-*b*-PHPMA triblock copolymers (blue).

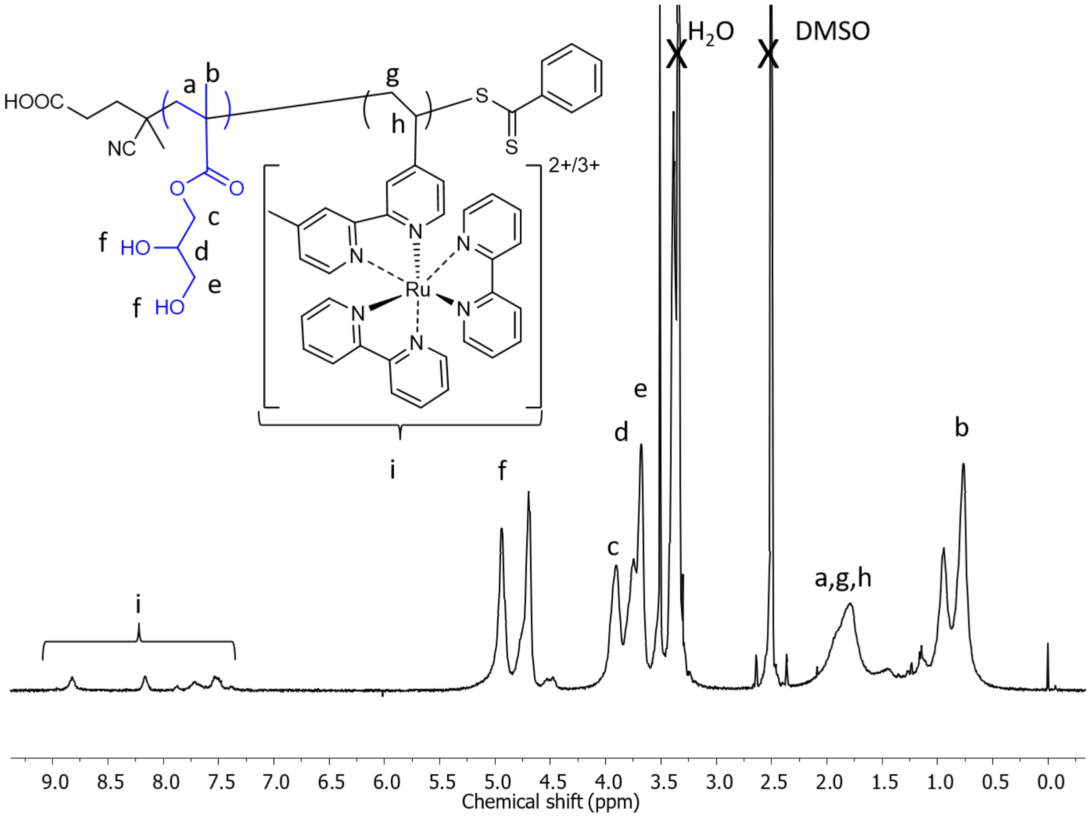

Figure S3. ^{1}H-NMR spectra of the PGMA-*b*-Ru(bpy)3 diblock copolymer in d6-DMSO.

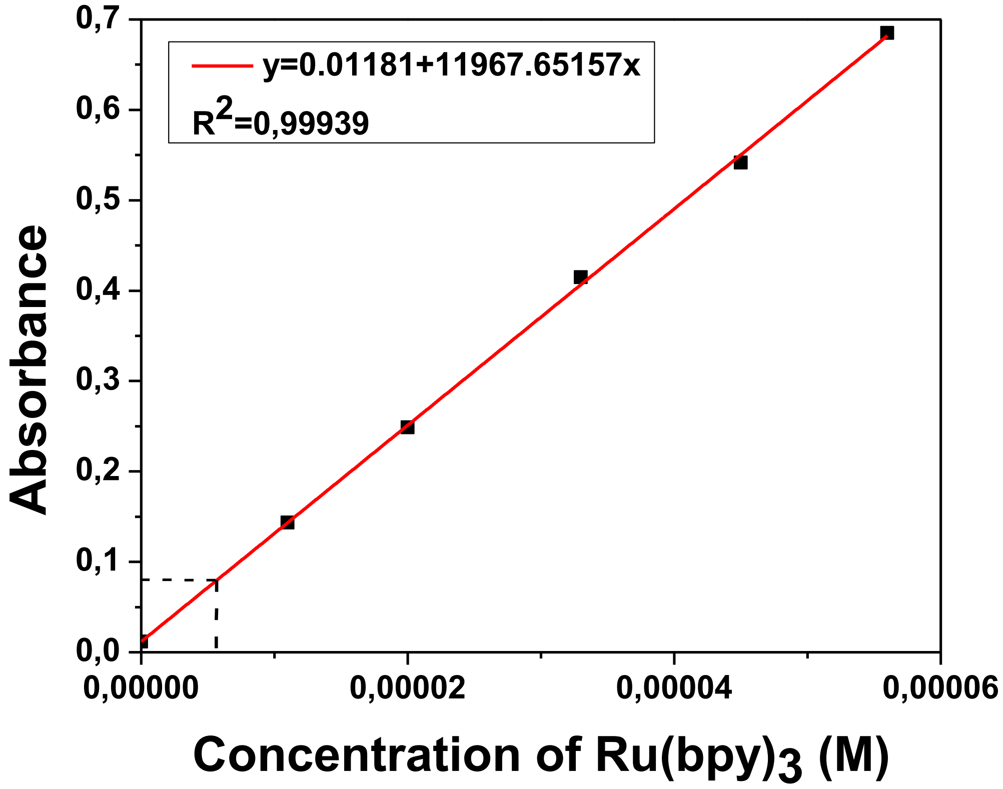

Figure S4. Calibration curve to determine the amount of Ru(bpy)3 conjugated to the polymer PGMA macro-CTA. The calibration curve was independently made from aqueous solutions of $Ru(bpy)_3PF_6$ with known concentrations.

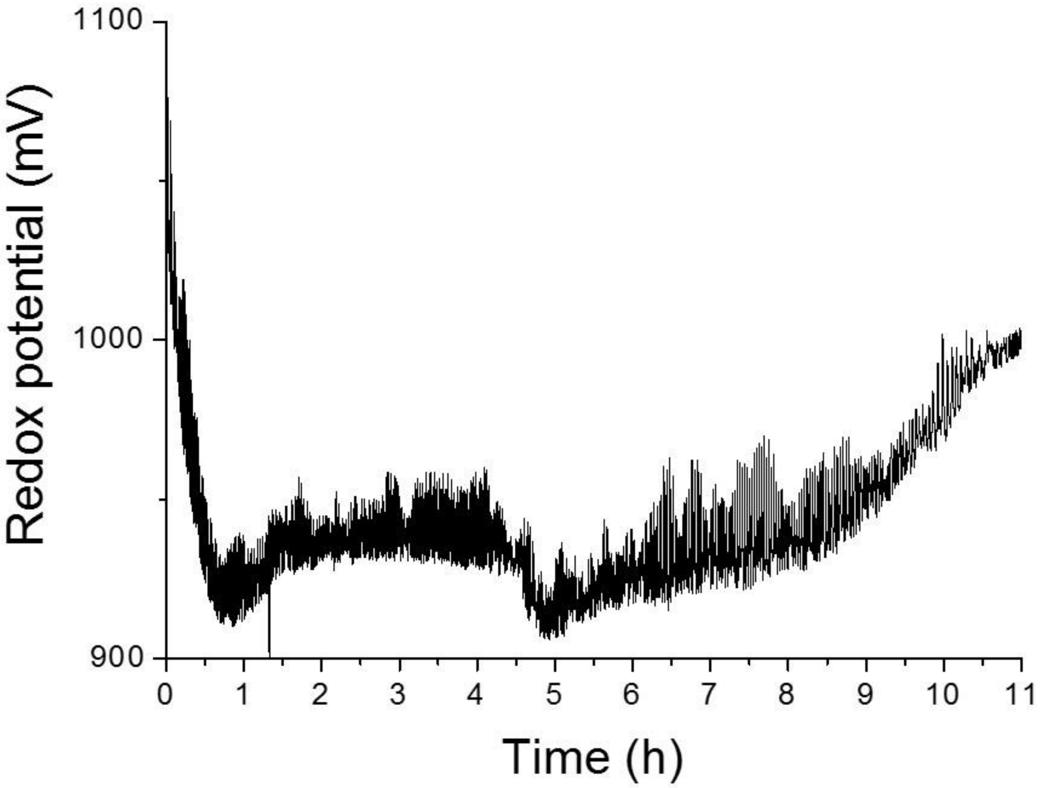

Figure S5. Oscillation profiles of redox potential at 20 ˚C for 0.5% *w/w* PGMA-*b*-Ru(bpy)$_3$ solutions. The solution contains MA (62.4 mM), $NaBrO_3$ (84 mM), and HNO_3 (300 mM).

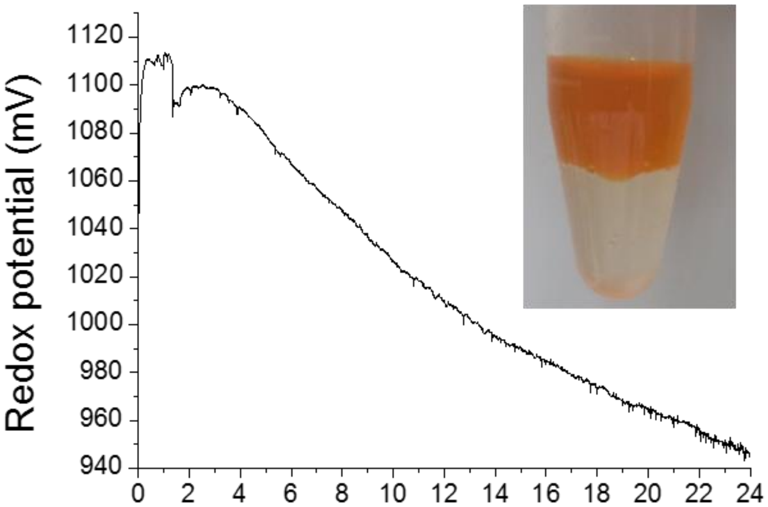

Figure S6. Oscillation profiles of redox potential for control experiment. The inset picture shows the resulting product after 24h.

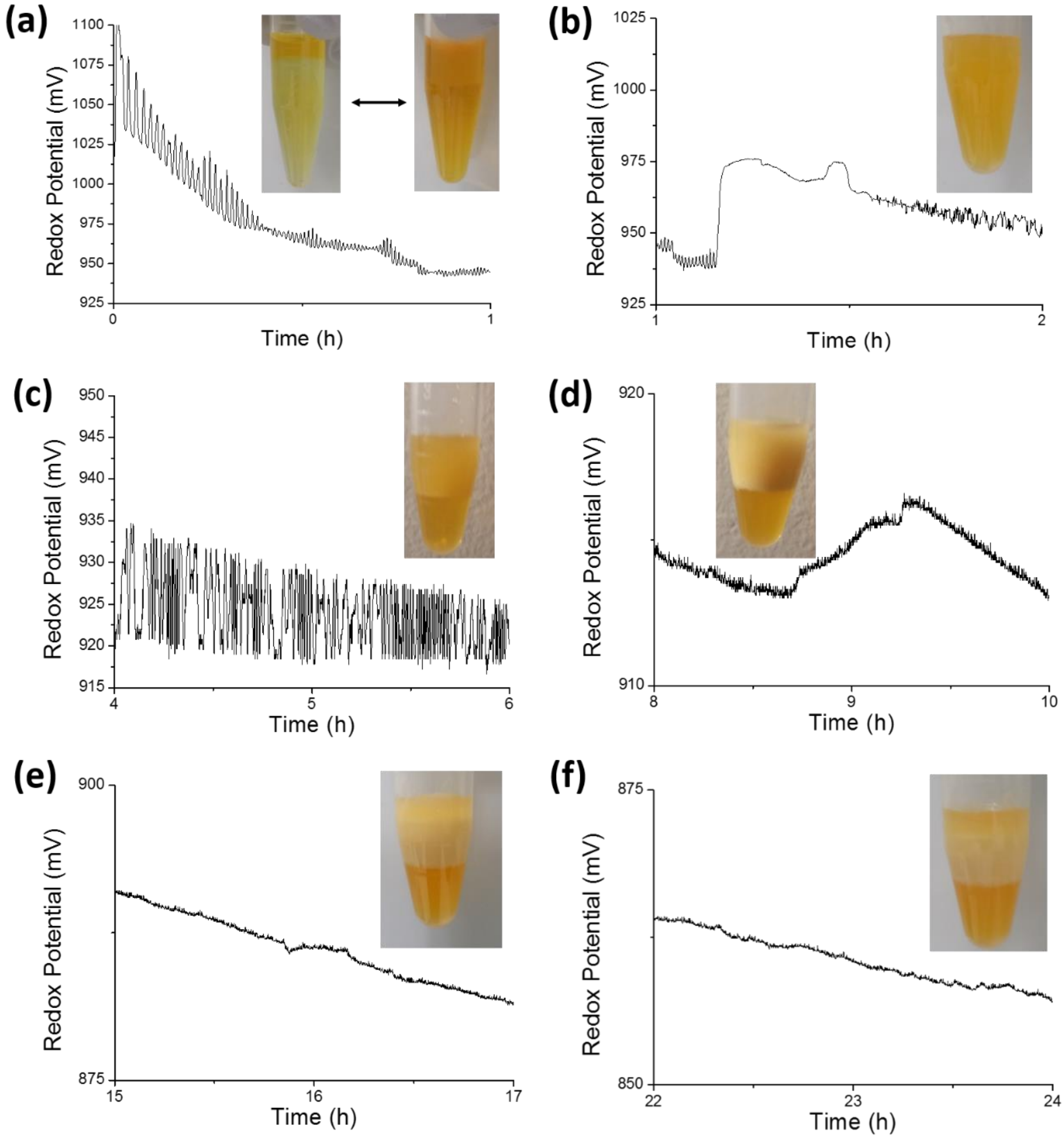

Figure S7. Oscillation profiles of redox potential for the synthesis of PGMA-*b*-Ru(bpy)$_3$-*b*-PHPMA under the BZ reaction at different times: (**a**) 0h, (**b**) 2h, (**c**) 5h, (**d**) 10h, (**e**) 17h, (**f**) 24h. The solution contains MA (62.4 mM), $NaBrO_3$ (84 mM), and HNO_3 (300 mM).

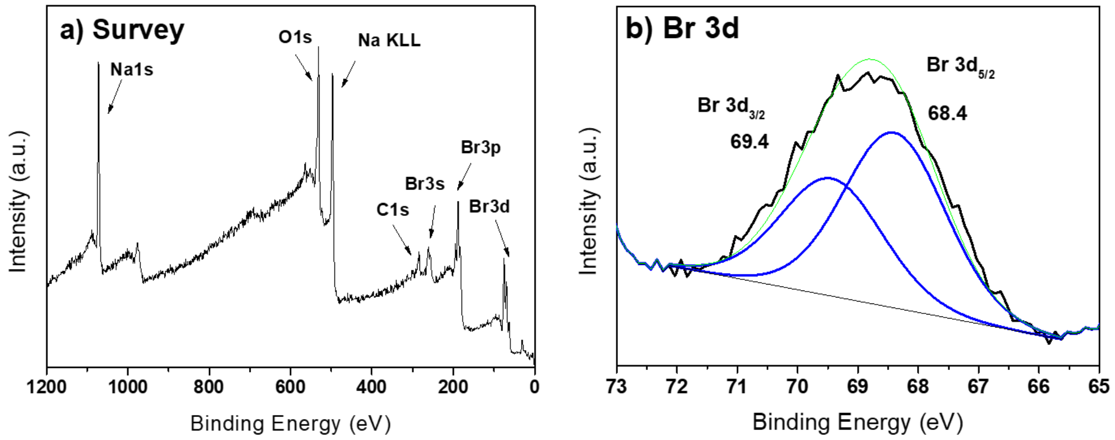

Figure S8. XPS spectra of $NaBrO_3$: A wide scan spectra (survey) (**a**) and Br 3d core level (**b**).

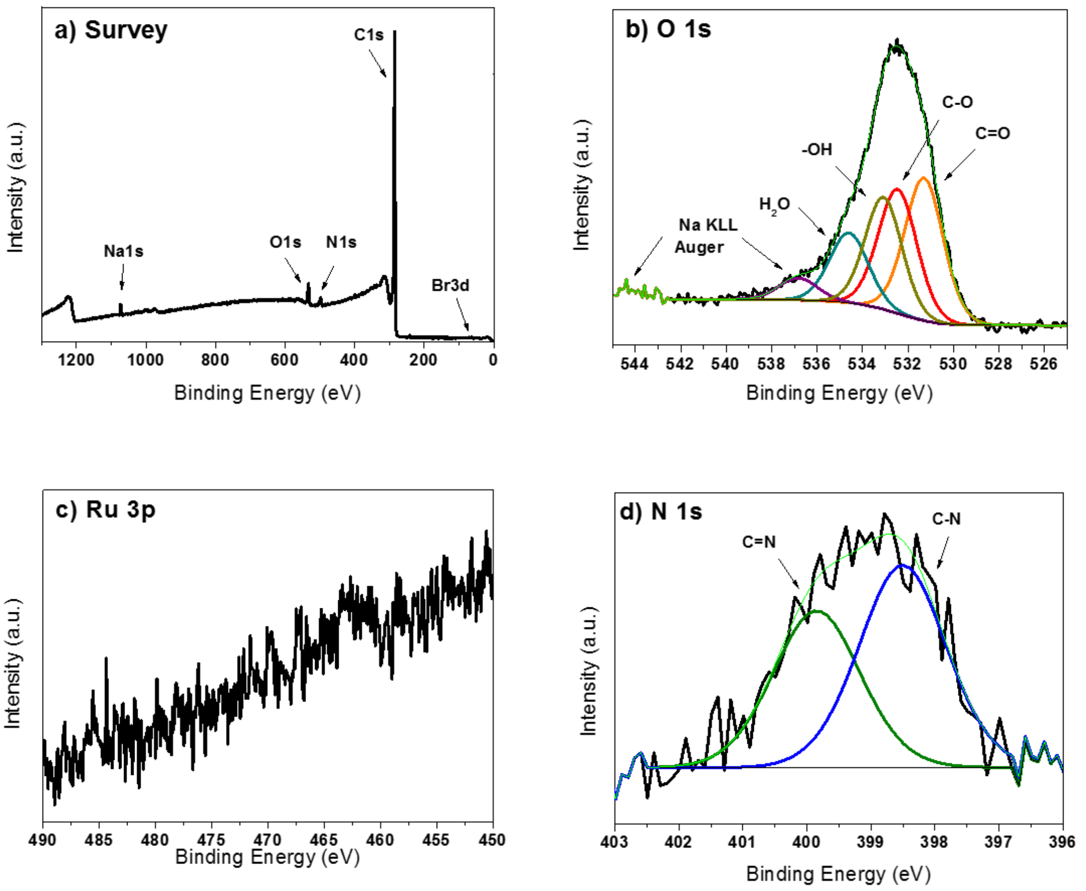

Figure S9. XPS spectra of a wide scan spectra (survey) (**a**), O 1s(**b**), Ru 3p(**c**), and N 1s (**d**), core level spectra of PGMA-*b*-Ru(bpy)$_3$-*b*-PHPMA triblock copolymer.

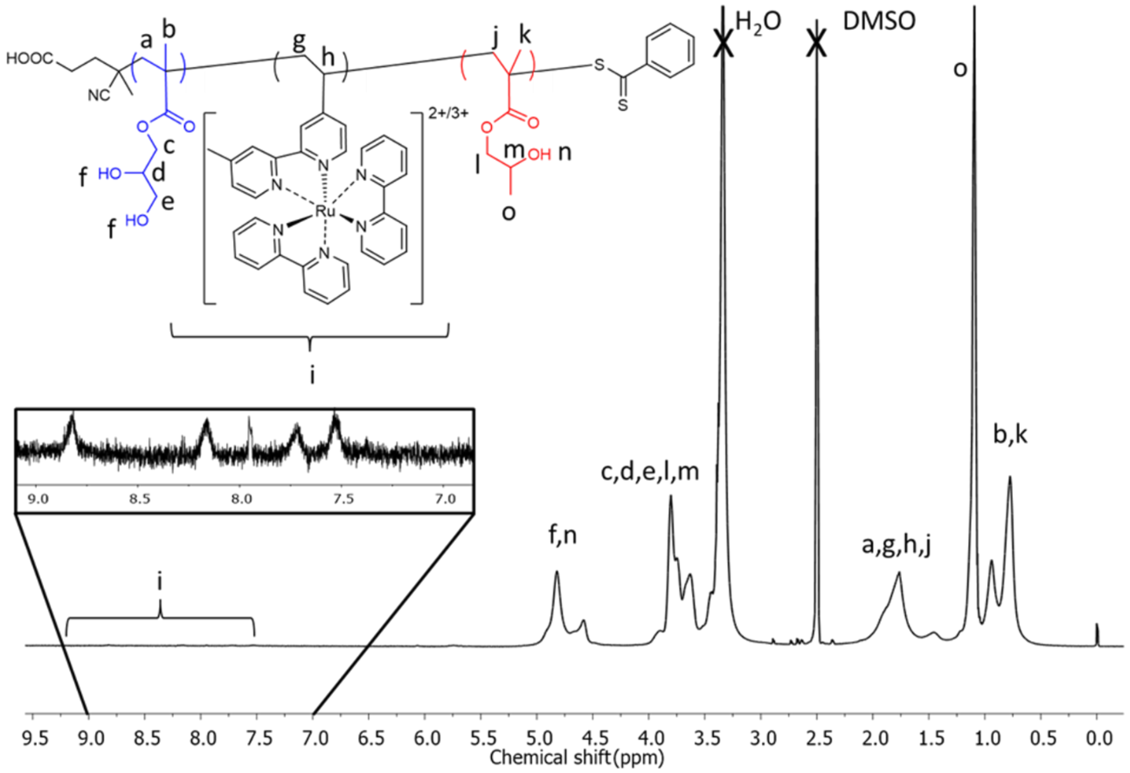

Figure S10. ^{1}H-NMR spectra of the PGMA-*b*-Ru(bpy)$_3$-*b*-PHPMA triblock copolymer in DMSO-d6.

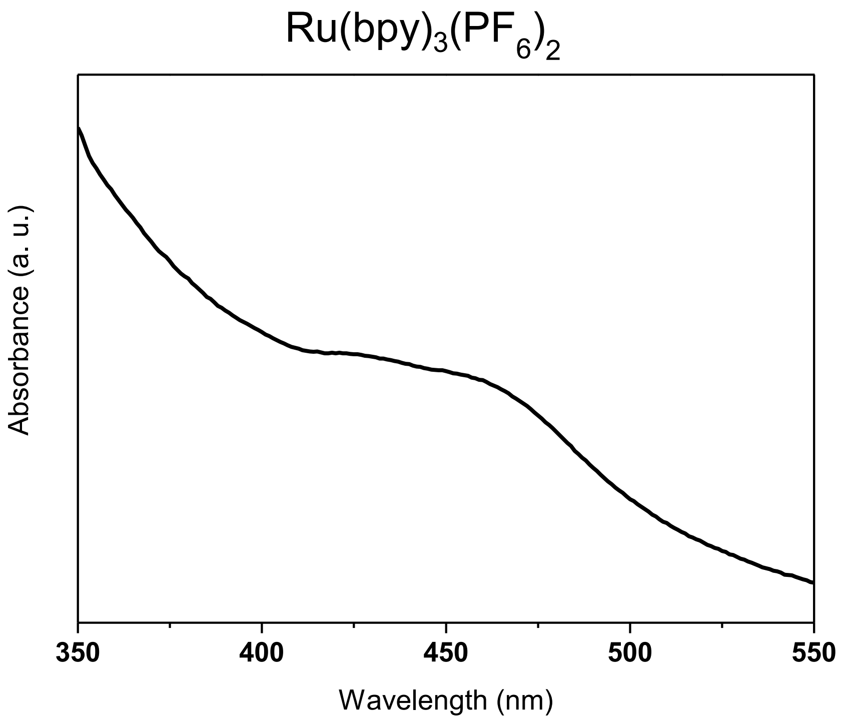

Figure S11. UV-visible absorption spectra of PGMA-*b*-Ru(bpy)$_3$-*b*-PHPMA triblock copolymer in a methanol solution.

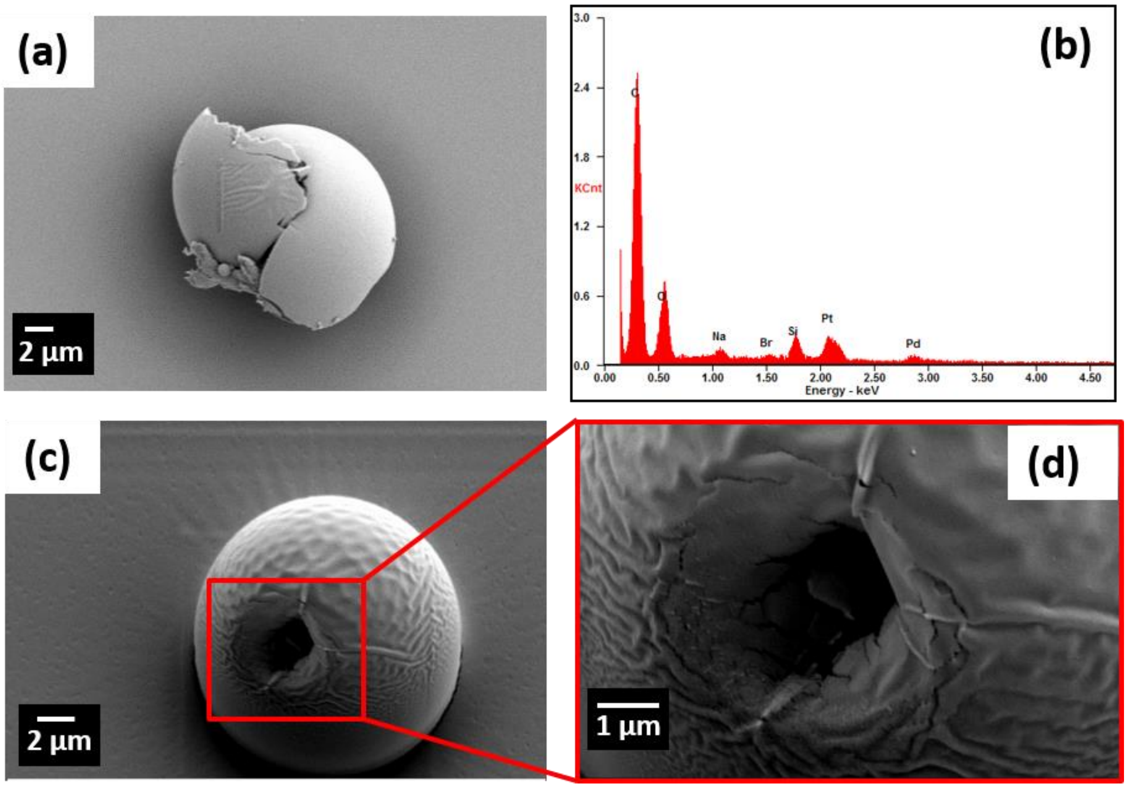

Figure S12. FE-SEM images of PGMA-*b*-Ru(bpy)$_3$-*b*-PHPMA triblock copolymer vesicles. A broken vesicle (**a**) with their corresponding EDXS analysis (**b**). A vesicle and a zoomed area shows a detail of the hollow that suggest the vesicle nature of this structure (**c**,**d**).

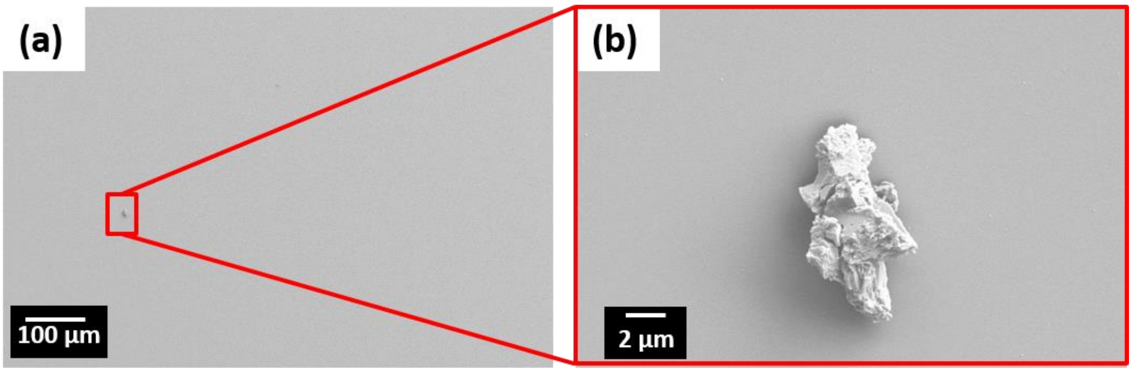

Figure S13. FE-SEM image of control experiment (**a**). A zoomed area shows a disorganized material (**b**).

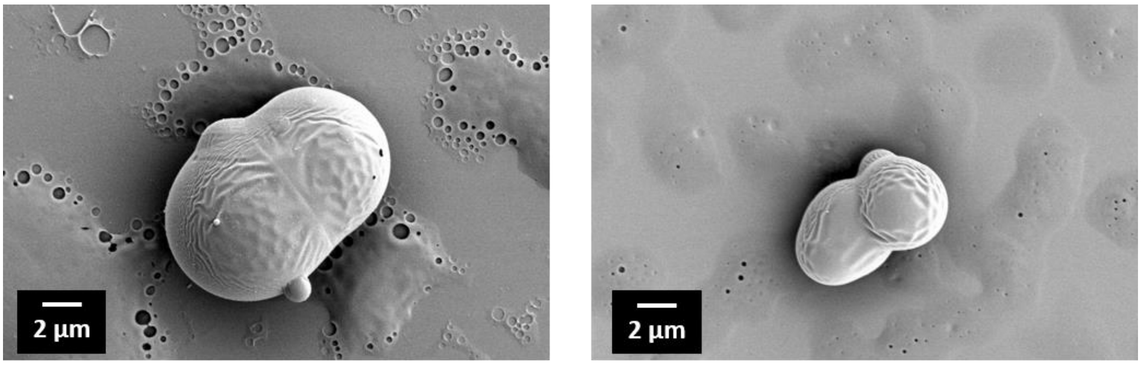

Figure S14. FE-SEM images shows examples of the morphology of fusing vesicles.

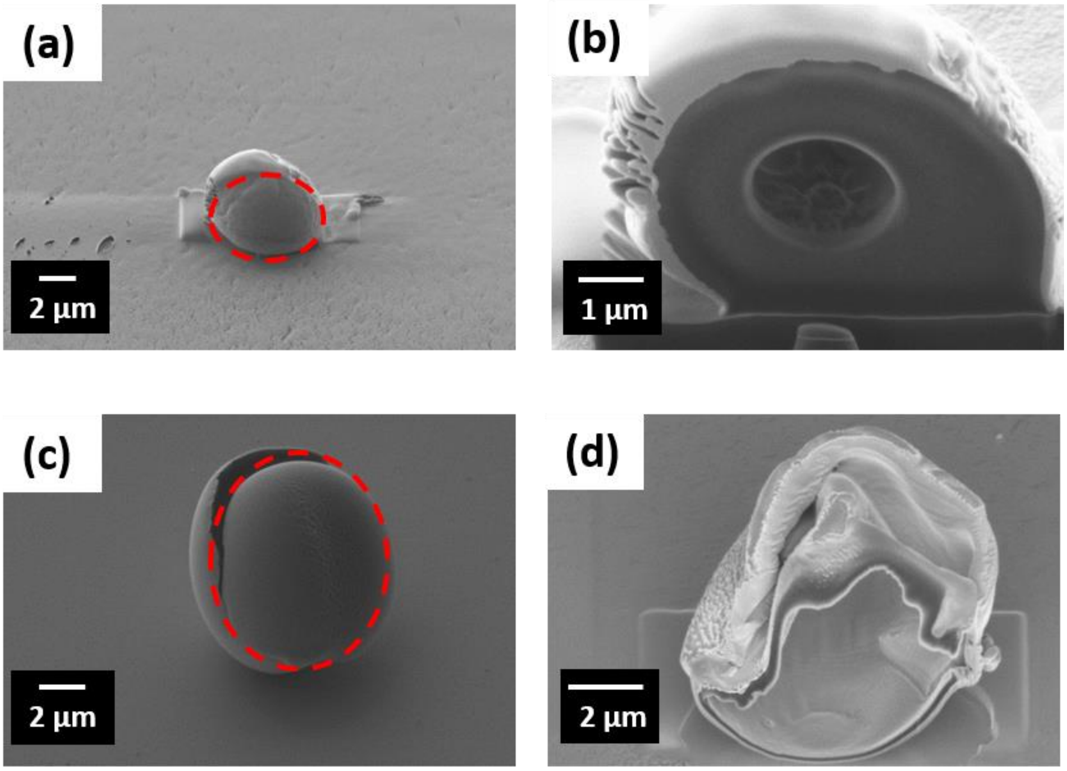

Figure S15. FE-SEM images of PGMA-*b*-Ru(bpy)$_3$-*b*-PHPMA triblock copolymer vesicles before (**a,c**) and after milling (**b**,**d**). The red circles indicate the portions where FIB milling was performed.

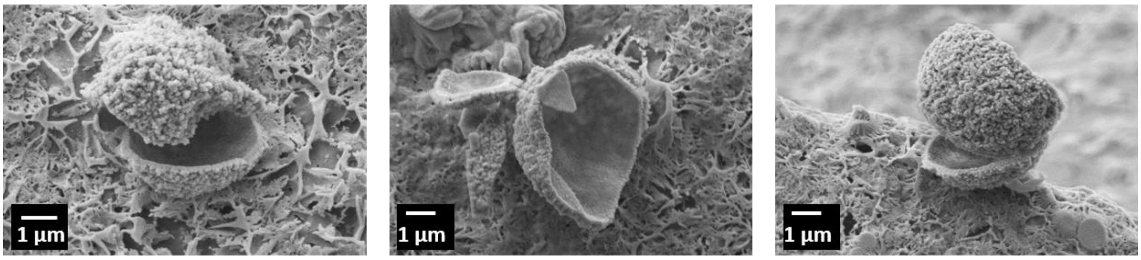

Figure S16. Cryo-SEM images of PGMA-*b*-Ru(bpy)$_3$-*b*-PHPMA triblock copolymer vesicles in a water/methanol mixture solution.
